# Assessing the Public’s Preferences and Outcomes in Using Online Resources and Digital Health Tools to Manage Skin Conditions: A Cross-Sectional Study

**DOI:** 10.7759/cureus.82188

**Published:** 2025-04-13

**Authors:** Fawwaz F Alshammari, Aala H Alhobera, Wijdan Alsaad, Rozan Alshammari

**Affiliations:** 1 Dermatology, University of Hail College of Medicine, Hail, SAU; 2 Dermatology, King Salman Specialist Hospital, Hail, SAU; 3 College of Medicine, University of Hail College of Medicine, Hail, SAU

**Keywords:** dermatology, digital tools, online resources, platforms, saudi arabia, skin conditions

## Abstract

Background

Individuals often seek advice from professionals and others to make decisions that best suit their needs in various situations. With the rapid growth of technology and the variety of available platforms, the general public faces the dilemma of choosing from numerous, diverse options. This study aims to explore the public's online advice-seeking behaviors, along with the reliability and trustworthiness of the information they use to manage their skin conditions.

Methods

A sample of 853 participants, aged 15 and above, from Saudi Arabia, completed a 20-question close-ended questionnaire, along with one optional open-ended question, assessing their use of online and digital resources for skin-related concerns.

Results

Statistical analysis revealed that the primary reason for seeking online solutions was convenience (170 participants, or 53.6%), followed by curiosity or self-education (133 participants, or 42%). A significant portion of respondents (164 participants, or 53%) found online information reliable, while 41 (12.9%) did not. Regarding skin condition improvement, 217 participants (68.4%) reported noticing a positive change, while four (1.3%) observed a decline.

Conclusion

This study highlights the importance of online resources and digital health tools for obtaining health-related information. A clear, positive correlation was found between the use of such resources and improvement in skin conditions.

## Introduction

The increasing use of online resources and digital health tools for managing skin conditions has become a significant trend globally. In Saudi Arabia, individuals are increasingly turning to digital platforms for advice on managing dermatological issues such as acne, eczema, and psoriasis. These platforms provide users with convenient access to information, but they also present challenges regarding the accuracy and reliability of the content. With the vast amount of health information available online, patients must navigate numerous sources to determine what is credible and beneficial for managing their skin conditions [1].

Research has shown that a growing number of individuals use social media and digital platforms as sources of dermatological advice. Platforms such as Instagram, TikTok, and YouTube have emerged as key tools for both professional dermatologists and influencers who provide advice on skin health. These platforms influence users' health-seeking behaviors, enabling individuals to self-assess and manage conditions before consulting healthcare professionals. Previous studies have found that social media use is particularly influential in shaping dermatological awareness and preventive behaviors among the public, with individuals often relying on these platforms to stay informed about skin care practices [2,3].

In addition to social media, digital health tools like teledermatology services and mobile apps offer new opportunities for managing skin conditions. While these tools hold the potential to improve access to dermatological care, their effectiveness in producing positive health outcomes is still a subject of investigation. Research indicates that, while some users report improvements in their conditions, others experience no change or even worsening symptoms, which highlights the mixed outcomes associated with the use of digital health resources [4,5]. Despite these mixed results, preferences for in-person consultations remain, particularly for more complex dermatological issues. This ongoing preference underscores the need for improvements in the accessibility, reliability, and trustworthiness of online health tools [6].

Furthermore, the role of artificial intelligence (AI) in dermatology is an emerging area of interest. AI-powered tools are beginning to assist dermatologists in diagnosing skin conditions and offering personalized treatment recommendations. However, patient perceptions of AI's role in healthcare remain divided. While some individuals see AI as a promising tool for enhancing diagnostic accuracy and treatment efficiency, others are skeptical about its ability to replace traditional in-person consultations and the human element of care [7]. As AI continues to evolve in dermatology, understanding its impact on patient care and its potential to improve health outcomes is crucial.

A growing concern with digital health tools is the tendency for individuals to self-medicate based on online health advice, which may lead to improper treatment or delayed diagnosis. Studies show that self-medication for skin conditions is common, particularly when individuals seek information on the internet before consulting a professional. This behavior is linked to the increasing use of online resources for diagnosing and treating minor ailments [8]. This study aims to assess the preferences of the public in Saudi Arabia regarding the use of online resources and digital health tools for managing skin conditions. Specifically, it explores how individuals decide whether to manage their conditions independently using online resources or seek professional dermatological care. The study also evaluates the outcomes individuals experience from using these tools, including improvements, no change, or worsening of their skin conditions. Additionally, the research investigates the factors that influence the use of digital health tools, such as trust, accessibility, and demographic variables like age and gender. By examining these factors, this study seeks to provide valuable insights into how online resources can be more effectively utilized in managing skin health and inform future healthcare strategies.

## Materials and methods

Objectives

This study aims to assess various aspects of online dermatologic information and examine public advice-seeking behaviors in Saudi Arabia. Specifically, it will evaluate factors such as the frequency of online resource usage, preferred formats, reasons for preference, perceived reliability of information, valued features, and the use of specific digital tools for skin management. The study will also explore obstacles encountered, reported improvements, and whether online resources are preferred over in-person consultations. Additionally, it will assess the likelihood of continued use of online sources and their expected role in the future. By analyzing these factors, the study aims to determine the public’s dependence on these resources and the associated outcomes.

Study design and sample

This cross-sectional study targeted both female and male individuals in Saudi Arabia who use online and digital resources to address skin-related concerns. The study was approved by the Research Ethics Committee at the University of Hail, Hail, Saudi Arabia (approval number: H-2024-508).

Data collection

Data were collected using an electronically administered questionnaire, available in both Arabic and English (Appendices). The questionnaire consisted of 20 closed-ended questions and one optional open-ended question. Data collectors were recruited from each region of Saudi Arabia and distributed the questionnaire via online platforms between October and December 2024. A convenience sampling method was employed, targeting the general public. The questionnaire was designed to filter participants who actively seek online and digital resources to address skin-related concerns across various regions of Saudi Arabia, making this the primary focus of the study.

Data analysis

Statistical analyses were conducted using IBM SPSS Statistics for Windows, Version 26 (Released 2019; IBM Corp., Armonk, NY, USA). Descriptive statistics, including frequencies and percentages, were employed to summarize the demographic and clinical characteristics of the study population. Categorical variables were analyzed using the Chi-square test to evaluate associations and identify statistically significant relationships between variables. When the expected cell count was <5 in 20% or more of the cells, Fisher's exact test was applied to ensure the accuracy of the results. Statistical significance was determined at a p-value threshold of <0.05.

## Results

Table 1 summarizes the demographic characteristics of the study sample, analyzed by age, sex, educational level, and region. A total of 853 participants were included in the study. Participants aged 20-25 years comprised 234 (27.4%) of the sample, followed by those aged 40-50 years (231, or 27.1%), 15-19 years (125, or 14.7%), 35-40 years (89, or 10.4%), 25-30 years (77, or 9.0%), 30-35 years (60, or 7.0%), and those over 50 years (37, or 4.3%). In terms of gender, 545 (63.9%) of the participants were female, while 308 (36.1%) were male. Regarding educational level, most participants (547, or 64.1%) held a bachelor’s degree. The remaining 181 (21.2%) participants had completed high school, 49 (5.7%) obtained a master’s degree, 29 (3.4%) completed secondary school, 28 (3.3%) had no formal education, 12 (1.4%) finished primary school, and seven (0.8%) were categorized as other. Geographically, the western region had the highest representation, with 265 (31.1%) participants, followed by the northern region (162, or 19.0%), the middle region (155, or 18.2%), the eastern region (154, or 18.1%), and the southern region (117, or 13.7%).

**Table 1:** Demographic characteristics of the study sample

Variables	Characteristics	Frequency	Percentage
Age	15-19 years	125	14.7
	20-25 years	234	27.4
	25-30 years	77	9.0
	30-35 years	60	7.0
	35-40 years	89	10.4
	40-50 years	231	27.1
	>50 years	37	4.3
Gender	Male	308	36.1
	Female	545	63.9
Level of education	Primary school	12	1.4
	Secondary school	29	3.4
	High school	181	21.2
	Bachelor’s degree	547	64.1
	Master’s degree	49	5.7
	Other	7	0.8
	None	28	3.3
Region	Northern region	162	19.0
	Western region	265	31.1
	Middle region	155	18.2
	Eastern region	154	18.1
	Southern region	117	13.7

Table 2 shows that 458 participants (56.9%) reported no skin conditions, while 368 (43.1%) reported having a skin condition. Of those with skin conditions, 317 (86.1%) frequently used the internet to seek information, compared to 51 (13.9%) who did not. Regarding the frequency of internet use for skin-related information, 34 (10.7%) searched daily, 55 (17.4%) searched weekly, 105 (33.1%) searched monthly, 121 (38.2%) searched rarely, and two (0.6%) searched never. These findings suggest a significant reliance on the internet for health information, with varying levels of engagement depending on individual needs and habits.

**Table 2:** Frequency of internet use and skin condition awareness

Variables	Characteristics	Frequency	Percentage
Do you have any skin conditions?	No	485	56.9
	Yes	368	43.1
Do you search the internet for information about your skin condition?	No	51	13.9
	Yes	317	86.1
If yes, how often do you use the internet to search for information about skin conditions?	Daily	34	10.7
	Weekly	55	17.4
	Monthly	105	33.1
	Rarely	121	38.2
	Never	2	0.6

**Figure 1:**
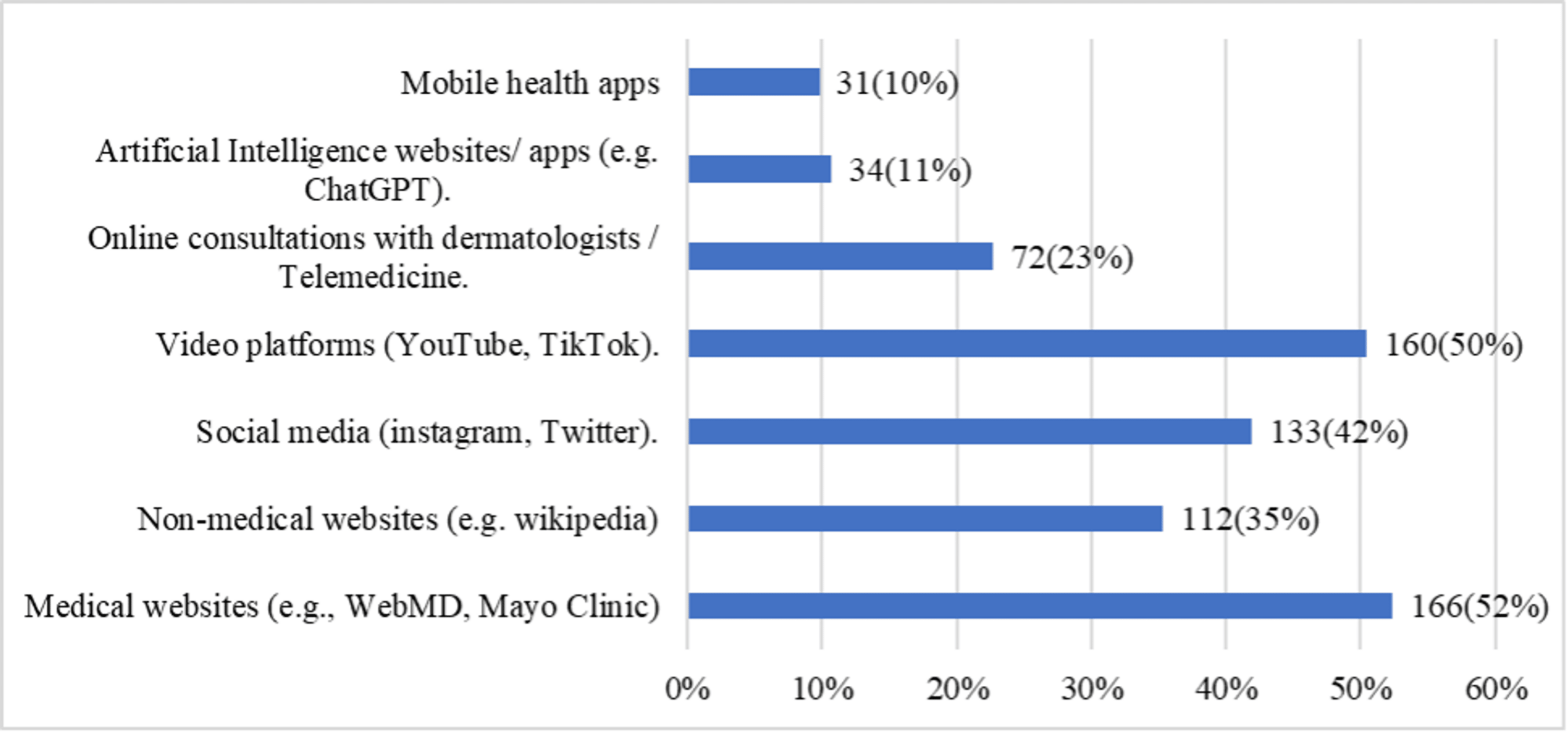
Types of online resources used (multiple choices)

Figure 1 shows the diverse range of online resources used to manage skin conditions. Medical websites were the most frequently used (166, or 52%), followed by video platforms (160, or 50%) and social media (133, or 42%). Non-medical websites accounted for 112 (35%) of the usage, whereas online consultations with dermatologists and telemedicine services were used by 72 (23%) of respondents. Mobile health apps were preferred by 31 (10%) participants, and AI-based websites or apps were the least used, at 34 (11%) participants. These findings indicate a diverse approach to managing skin conditions online, with significant reliance on both medical and non-medical resources.

**Figure 2:**
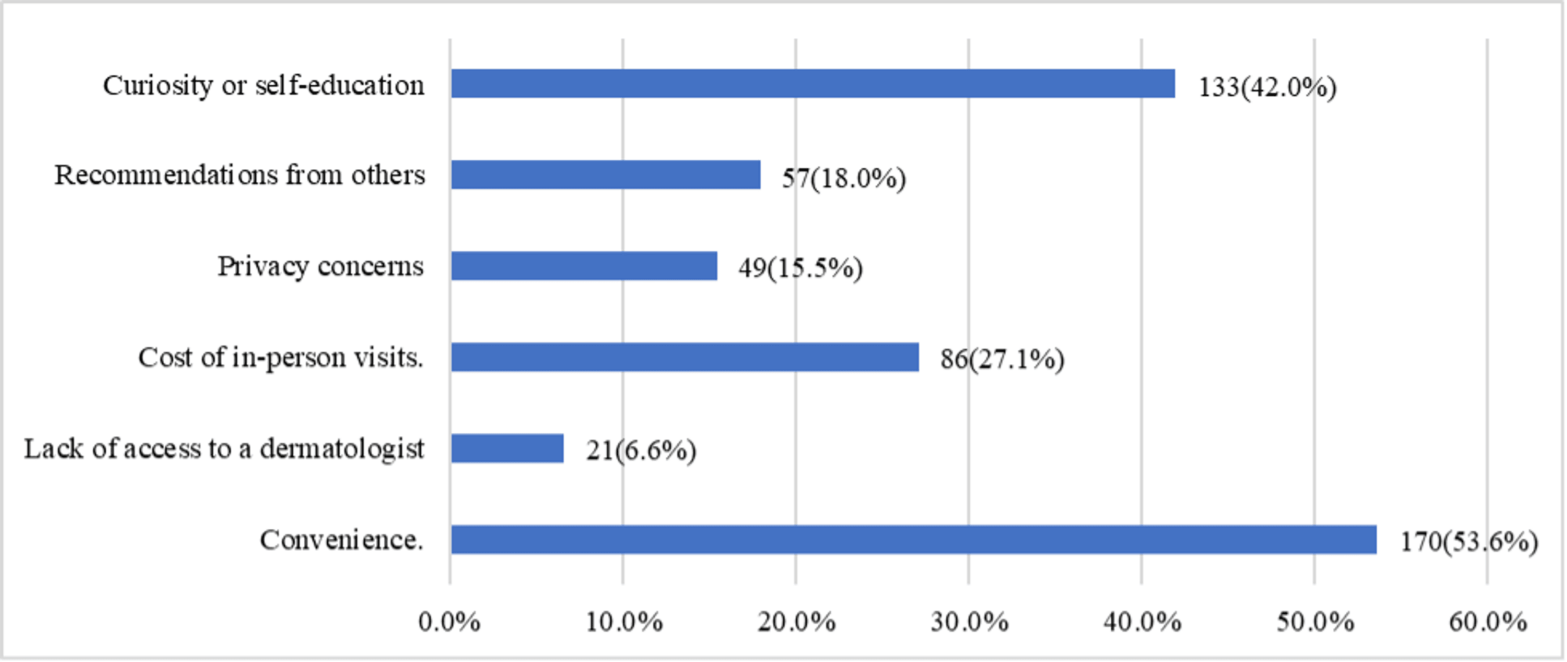
Reasons for using online resources to address skin conditions (multiple choices)

Figure 2 illustrates the diverse motivations for using online resources to manage skin conditions. Convenience was the most common reason (170, or 53.6%), followed by curiosity or self-education (133, or 42.0%), and cost considerations, particularly the expense of in-person visits (86, or 27.1%), while 57 (18.0%) were influenced by recommendations. Privacy concerns drove 49 (15.5%) individuals to use these resources, and a lack of access to a dermatologist influenced 21 (6.6%) participants. These findings highlight the diverse motivations behind using digital tools for skin health management.

When assessing the reliability of online resources, 141 (44.5%) of participants considered them somewhat reliable, 108 (34.1%) were neutral, 38 (12%) found them somewhat unreliable, 27 (8.5%) viewed them as very reliable, and three (0.9%) considered the information very unreliable. To evaluate the reliability of online information, 108 (34.1%) cross-checked information across multiple sources, 71 (22.4%) trusted reputable medical websites, 58 (18.3%) considered the information reliable if shared by professionals, 49 (15.5%) relied on peer reviews or ratings, and 31 (9.8%) found it difficult to assess. Regarding the use of digital tools for managing skin conditions, 191 (60.3%) of the participants had not used any tools, whereas 126 (39.7%) had. Of those, 79 (24.9%) had used mobile apps for skin tracking. Additionally, 51 (16.1%) used teledermatology services, 38 (12%) used wearable devices, and 13 (4.1%) relied on AI-powered skin analysis tools (Table 3).

**Table 3:** Preceptive reliability of online resources and digital tools in addressing skin conditions

Variables	Characteristics	Frequency	Percentage
How reliable do you find the information on these online resources?	Very reliable	27	8.5
	Somewhat reliable	141	44.5
	Neutral	108	34.1
	Somewhat unreliable	38	12.0
	Very unreliable	3	0.9
How do you assess the reliability of the information you find online?	I trust reputable medical websites only	71	22.4
	I cross-check information across multiple sources	108	34.1
	I rely on peer reviews or ratings	49	15.5
	I consider information reliable if it’s shared by professionals	58	18.3
	I find it difficult to assess the reliability	31	9.8
Have you used any digital tools specifically designed for managing skin conditions (e.g., health apps, telemedicine platforms)?	No	191	60.3
	Yes	126	39.7
If yes, which tools have you used? (multiple choices)	Mobile apps for skin tracking	79	24.9%
	Teledermatology services	51	16.1%
	Wearable devices	38	12.0%
	AI-powered skin analysis tools	13	4.1%

**Figure 3:**
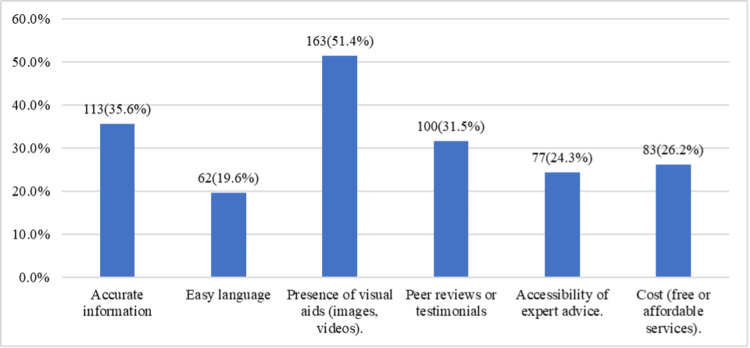
Features considered in online resources for skin condition management (multiple choices)

Figure 3 highlights the features considered most valuable in online resources for skin condition management. The presence of visual aids, such as images and videos, was the most important feature for 163 (51.4%) participants, followed by accurate information for 113 (35.6%) and peer reviews or testimonials for 100 (31.5%). Cost, particularly free or affordable services, was highlighted by 83 (26.2%), and 77 (24.3%) prioritized access to expert advice. Easy-to-understand language was valued by 62 (19.6%). These results suggest that visual aids, accuracy, and peer feedback are key factors in selecting online resources for skin care.

**Figure 4:**
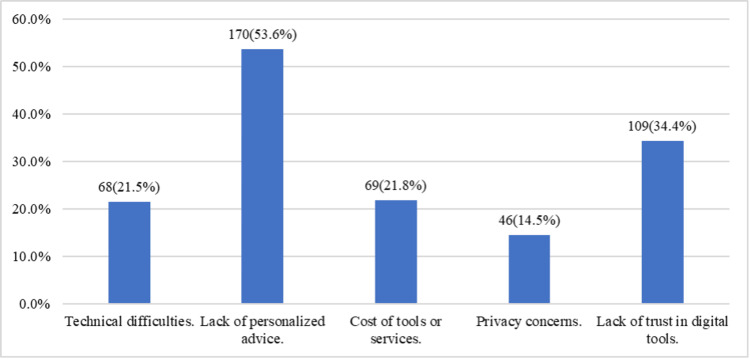
Challenges faced in using digital tools and online resources for managing skin conditions (multiple choices)

Figure 4 illustrates the challenges participants faced when using online sources and digital health tools to manage skin conditions. Lack of personalized advice was the most common concern for 170 (53.6%) participants, followed by a lack of trust in digital tools for 109 (34.4%) participants. Technical difficulties were reported by 68 (21.5%), and 46 (14.5%) expressed privacy concerns. Additionally, 69 (21.8%) were concerned about the costs of tools or services. These findings indicate that, while digital health tools offer convenience, users face significant barriers in terms of personalization, trust, and technical reliability.

Table 4 presents the impact of online resources and digital tools on skin condition management. While 156 (49.2%) participants reported some improvement and 61 (19.2%) experienced significant improvement, 96 (30.3%) noted no change, and four (1.3%) reported slight worsening of their condition. Regarding recommendations, 44 (13.9%) respondents were definitely positive, with 126 (39.7%) generally recommending these tools. However, when asked about their preference for consultations, 31 (9.8%) strongly preferred online consultations, 58 (18.3%) somewhat preferred online, and 55 (17.4%) had no preference, while 84 (26.5%) somewhat preferred in-person consultations, and 89 (28.1%) strongly preferred in-person consultations. Regarding future usage of digital tools, 64 (20.2%) expressed the likelihood of continuing, with 123 (38.8%) being somewhat likely, and 81 (25.6%) being neutral. Finally, 208 (65.6%) believed that digital health tools and AI would play a larger role in managing skin conditions in the future, 73 (23.0%) were unsure, and 36 (11.4%) disagreed. These findings suggest that, while digital tools are perceived as helpful, there remains a strong preference for in-person care and a degree of skepticism regarding the full integration of AI into skin health management.

**Table 4:** Participant-reported outcomes, attitudes and future role of digital tools in dermatological care

Variables	Characteristics	Frequency	Percentage
How has the use of online resources or digital tools impacted your skin condition management?	Significant improvement	61	19.2
	Some improvement	156	49.2
	No change	96	30.3
	Slight worsening	4	1.3
Would you recommend online resources or digital tools to others for managing skin conditions?	Definitely	44	13.9
	Probably	126	39.7
	Not sure	98	30.9
	Probably not	36	11.4
	Definitely not	13	4.1
Do you prefer online or in-person consultations for managing skin conditions?	Strongly prefer online	31	9.8
	Somewhat prefer online	58	18.3
	No preference	55	17.4
	Somewhat prefer in-person	84	26.5
	Strongly prefer in-person	89	28.1
How likely are you to continue using online resources or digital tools for skin condition management in the future?	Very likely	64	20.2
	Somewhat likely	123	38.8
	Neutral	81	25.6
	Somewhat unlikely	40	12.6
	Very unlikely	9	2.8
Do you think digital health tools and artificial intelligence will play a larger role in managing skin conditions in the future?	No	36	11.4
	Yes	208	65.6
	Unsure	73	23.0

The results of the Chi-square test in Table 5 revealed a significant association between sex and online searches for skin conditions. Among men, 20 (20.6%) reported searching frequently, 77 (79.4%) reported searching occasionally, and zero (0%) reported never searching, while 69 (31.4%) women reported searching frequently, 149 (67.7%) reported occasionally, and two (0.9%) reported never (χ² = 4.539, p = 0.023). A significant relationship was also observed between age and online search behaviors. Specifically, 42 (29.8%) individuals aged 18-25 years reported searching frequently, 97 (68.8%) reported searching occasionally, and two (1.4%) reported never; 27 (32.5%) individuals aged 25-40 years reported searching frequently, 56 (67.5%) reported searching occasionally, and zero (0%) reported never; and 20 (21.5%) individuals aged over 40 years reported searching frequently, 73 (78.5%) reported occasionally, and zero (0%) reported never (χ² = 4.731, p = 0.036). These findings suggest that sex and age play crucial roles in determining patterns of online health information-seeking behaviors related to skin conditions.

**Table 5:** Relationship between gender and age and online search behavior in addressing skin conditions

Variables	Characteristics	Online search for skin conditions			χ2	p-value
		Frequently	Occasionally	Never		
Gender	Male	20 (20.6%)	77 (79.4%)	0 (0%)	4.539	0.023
	Female	69 (31.4%)	149 (67.7%)	2 (0.9%)		
Age	18-25 years	42 (29.8%)	97 (68.8%)	2 (1.4%)	4.731	0.036
	25-40 years	27 (32.5%)	56 (67.5%)	0 (0%)		
	Above 40 years	20 (21.5%)	73 (78.5%)	0 (0%)		

The impact of online tools on skin condition management was significantly influenced by participants' engagement with the internet. The results presented in Table 6 show that frequent users of online resources were more likely to experience improvement (69, or 77.5%), compared to occasional users (147, or 65%) or non-users (1, or 50%) (χ² = 9.444, p = 0.040). Furthermore, 150 (80.2%) of those likely to continue using digital tools in the future reported improvement, compared with only 21 (42.9%) of those unlikely to continue (χ² = 34.177, p = 0.001). These findings highlight the positive impact of online tools on skin condition management.

**Table 6:** Relationship between the impact of online tools on skin condition management and users’ behaviors and perceptions

Variables	Characteristics	Impact of online tools on skin condition management			χ2	p-value
		Improvement	No change	Worsening		
How often do you use the internet to search for information about skin conditions?	Frequently	69 (77.5%)	18 (20.2%)	2 (2.2%)	9.444	0.040
	Occasionally	147 (65%)	77 (34.1%)	2 (0.6%)		
	Never	1 (50%)	1 (50%)	0 (0%)		
How likely are you to continue using online resources or digital tools for skin condition management in the future?	Likely	150 (80.2%)	34 (18.2%)	3 (1.6%)	34.177	0.001
	Neutral	46 (56.8%)	34 (42%)	1 (1.2%)		
	Unlikely	21 (42.9%)	28 (57.1%)	0 (0%)		

The participants were asked to share their opinions on potential improvements to online resources or digital tools for managing skin conditions. They emphasized a strong need for reliable, trustworthy, and accurate online platforms to address skin-related issues effectively. Key suggestions include direct and easy access to specialized dermatologists through video calls or chat platforms, allowing for the sharing of photos for precise diagnosis. They also emphasized the importance of credible, medically reviewed information from qualified sources, free from commercial bias or misleading promotions. Patient experiences and testimonials should be documented to help others relate and learn. Privacy, quick responses, and a user-friendly interface were also highlighted as essential. Additionally, integrating AI and advanced technologies for accurate assessment and personalized treatment plans was suggested, along with expanding health awareness campaigns led by certified experts.

## Discussion

This study evaluated the extent to which online information on skin conditions meets the needs of individuals seeking dermatological advice in Saudi Arabia. It explored how accessible and reliable online resources influence individuals' decisions regarding whether to consult a dermatologist or manage their skin conditions independently. The findings underscore the significant role that online resources and digital health tools play in managing skin conditions. Notably, 317 (86.1%) of individuals with skin conditions actively sought online resources, highlighting a clear inclination toward digital platforms for health-related information.

This observation aligns with previous work, which observed a notable number of individuals using the internet to research their dermatological conditions [9]. Social media platforms, such as Snapchat, TikTok, and Instagram, have also emerged as key sources for dermatological advice, with research highlighting their widespread use [10]. This trend is particularly evident among younger populations, as findings suggest that 83% rely on the internet as their primary source of medical information, demonstrating that the digital landscape is reshaping how individuals approach dermatological care [11].

The study revealed that convenience, the desire for self-education, cost, privacy concerns, and limited access to dermatologists motivate the use of online resources. However, only 27 (8.5%) of participants deemed these resources highly reliable, while 141 (44.5%) found them somewhat reliable. This echoes previous studies, which observed that many individuals prefer online consultations or self-assessments before seeking professional help [12]. Other research further explored this labyrinth of challenges, highlighting difficulties concerning the availability and allocation of resources, financial aspects, reliability, and security [13]. Similarly, this skepticism shows that patients are more likely to trust online health information if it is authored by a doctor or a reputable health institution. This suggests that, despite the accessibility of online health resources, individuals still view medical professionals as their primary and most trusted source of information, even when they engage in online self-assessment or consultations [14]. Despite the availability of digital tools such as skin tracking apps (79, or 27.9%) and teledermatology services (51, or 16.1%), 191 (60.3%) participants did not utilize them, emphasizing the need for greater trust and enhanced accessibility.

The results underscore a fascinating yet enigmatic mosaic of outcomes when it comes to using online resources and digital tools for skin condition management. Among the participants, 217 (68.4%) reported improvements, 96 (30.3%) observed no change, and four (1.3%) experienced worsening conditions. The results show that 170 (53.6%) of respondents were positive about recommending these tools, yet 173 (54.6%) preferred in-person consultations, with 187 (59%) indicating a likelihood of continuing the use of digital tools. Furthermore, 208 (65.6%) believed AI would play an increasingly larger role in skin condition management, though there was evident skepticism, with 73 (23%) unsure and 36 (11.4%) disagreeing. These findings resonate with the suggestion that AI has the potential to assist dermatologists in identifying rare skin conditions and managing chronic diseases [15]. Similarly, the convenience of digital tools has been acknowledged, despite concerns about diagnostic precision and infrastructure limitations [16]. Other studies emphasized that, while digital health tools can certainly enhance healthcare access, barriers such as limited internet connectivity and unfamiliarity with digital tools must be transcended for effective implementation [17].

The demographic influences on online health information-seeking behaviors were also explored. The findings align with previous studies that reported women are more likely to engage in online health information-seeking behaviors, which can be attributed to their generally higher concern for health matters, driving them to delve deeper into available online resources [18,19]. Age also played a significant role, with younger adults (18-25 years) and middle-aged individuals (25-40 years) reporting higher levels of engagement, as younger adults are generally more familiar with digital tools and more inclined to seek information online [20].

Moreover, the positive relationship between internet use and improvements in skin conditions mirrors findings that suggest seeking health information online can enhance health literacy, potentially leading to better outcomes [21].

This study also highlighted the empowering role of online resources, enabling individuals to make informed decisions regarding their skin health, which can lead to improved management and positive outcomes. Frequent users of online resources experienced the most significant improvements (66, or 77.5%), reinforcing the importance of sustained engagement with digital tools. This continuous engagement seems to intertwine with a more proactive approach to self-management, allowing users to reimagine their skincare practices based on the information they access. Furthermore, 150 (80.2%) of individuals intending to continue using online tools reported improvements in their skin conditions, emphasizing the long-term benefits of digital health tools in personal healthcare routines. In contrast, only 21 (42.9%) of those who were unlikely to continue using digital tools experienced improvements, suggesting that the consistent use of these tools is essential for their effectiveness. The findings echo research that noted high engagement and retention rates among patients using a digital health program for atopic dermatitis, with improvements in symptoms and quality of life [22]. Further studies explored the role of AI-powered applications in enhancing patients' understanding of skin conditions, leading to better adherence and proactive management [23].

An additional study discussed the significance of consistent use for the effectiveness of digital health tools, which corresponds with the findings of this study that frequent users of online resources experience notable improvements in managing skin conditions [24]. Together, these studies demonstrate the critical role of engagement with digital tools in enhancing skin health management. The results provide strong justification for the role of online resources in managing skin conditions and highlight the potential for digital health tools to be an essential part of healthcare strategies for individuals seeking better skin health outcomes. The findings suggest that promoting digital literacy and continued engagement with online resources could significantly enhance the effectiveness of skin condition management, particularly among those who are already inclined to use these tools.

Limitations and future research

This study has several limitations that should be addressed in future research. First, the reliance on self-reported data may introduce biases, as participants' perceptions of online resources may not always align with objective measures of effectiveness. Additionally, the study primarily focused on individuals in the Kingdom of Saudi Arabia, limiting the generalizability of the findings to other regions or cultural contexts. Future research could expand to include more diverse populations and explore regional differences in digital health tool usage. The study also did not specify particular types of skin diseases, which could impact the interpretation of results, as individuals may seek digital resources for different skin concerns. Future research could benefit from categorizing skin diseases to better understand how digital tools address specific conditions. Moreover, the study did not assess the long-term outcomes of using digital tools, which warrants further investigation to understand the sustainability of benefits over time. Further studies should also explore the impact of specific types of online resources, such as mobile apps or teledermatology, on skin condition management and examine how AI and personalization in digital health tools can improve trust and reliability. Lastly, investigating the barriers to adopting digital tools among individuals less likely to use them could provide valuable insights for enhancing accessibility and effectiveness.

## Conclusions

This study underscores the pivotal role of online resources and digital health tools in managing skin conditions, particularly in Saudi Arabia. The findings revealed a strong preference for using the internet for health-related information, with a notable proportion of individuals actively seeking online resources for skin condition management. However, concerns remain regarding the reliability and trustworthiness of these resources, emphasizing the need for improved accuracy and accessibility. The positive relationship between online resource use and skin condition improvement supports the potential benefits of digital health tools in empowering individuals to effectively manage their skin health. Despite a preference for in-person consultations, integrating digital tools, including AI, may play a crucial role in future dermatological care. These results emphasize the importance of fostering digital literacy and continuous engagement with online resources to enhance health outcomes. This study highlights the critical role of online resources and digital health tools in managing skin conditions, emphasizing the importance of digital literacy and its impact on health outcomes. It reveals that frequent use of online platforms improves skin conditions, with younger and female individuals showing higher engagement. While digital tools are beneficial, the findings underscore the need for trustworthy, accessible, and tailored solutions, alongside the continued preference for traditional in-person consultations, advocating for integrated healthcare approaches.

## Appendices

MCQ Format Questionnaire (English version)

Q1) Gender

·      Male

·      Female

Q2) Age

·      15-19

·      20-25

·      25-30

·      30-35

·      35-40

·      40-50

·      50

Other…

Q3) Level of Education

·      Primary school

·      Secondary school

·      High school

·      Bachelor’s degree

·      Master’s degree

·      None

Other…

Q4) Do you have any skin conditions?

·      Yes

·      No

Q5) Location of Residence in Saudi Arabia (please specify your region below)

·      Northern region

·      Western region

·      Middle region

·      Eastern region

·      Southern region

Other…

Q6) Do you search the internet for information about your skin condition?

·      Yes

·      No

Q7) If yes, how often do you use the internet to search for information about skin conditions?

·      Daily

·      Weekly

·      Monthly

·      Rarely

·      Never

Q8) What type of online resources do you primarily use to manage skin conditions? (Select all that apply)

·      Medical websites (e.g., WebMD, Mayo Clinic)

·      Non-medical websites (e.g. Wikipedia)

·      Social media (Instagram, Twitter)

·      Video platforms (YouTube, TikTok)

·      Online consultations with dermatologists/Telemedicine

·      Artificial Intelligence websites/apps (e.g. ChatGPT)

·      Mobile health apps

Other…

Q9) Why do you use online resources for managing skin conditions? (Select all that apply)

·      Convenience

·      Lack of access to a dermatologist

·      Cost of in-person visits

·      Privacy concerns

·      Recommendations from others

·      Curiosity or self-education

Q10) How reliable do you find the information on these online resources?

·      Very reliable

·      Somewhat reliable

·      Neutral

·      Somewhat unreliable

·      Very unreliable

Q11) How do you assess the reliability of the information you find online?

·      I trust reputable medical websites only

·      I cross-check information across multiple sources

·      I rely on peer reviews or ratings

·      I consider information reliable if it’s shared by professionals

·      I find it difficult to assess reliability

Q12) What features do you value most in online resources for skin condition management? (Select all that apply)

·      Accurate information

·      Easy language

·      Presence of visual aids (images, videos)

·      Peer reviews or testimonials

·      Accessibility of expert advice

·      Cost (free or affordable services)

Q13) Have you used any digital tools specifically designed for managing skin conditions (e.g., health apps, telemedicine platforms)?

·      Yes

·      No

Q14) If yes, which tools have you used? (Select all that apply)

·      Mobile apps for skin tracking

·      Teledermatology services

·      Wearable devices

·      AI-powered skin analysis tools

Other…

Q15) What challenges have you faced in using online sources and digital health tools for the management of skin conditions? (Select all that apply)

·      Technical difficulties

·      Lack of personalized advice

·      Cost of tools or services

·      Privacy concerns

·      Lack of trust in digital tools

Other…

Q16) How has the use of online resources or digital tools impacted your skin condition management?

·      Significant improvement

·      Some improvement

·      No change

·      Slight worsening

·      Significant worsening

Q17) Would you recommend online resources or digital tools to others for managing skin conditions?

·      Definitely

·      Probably

·      Not sure

·      Probably not

·      Definitely not

Q18) Do you prefer online or in-person consultations for managing skin conditions?

·      Strongly prefer online

·      Somewhat prefer online

·      No preference

·      Somewhat prefer in-person

·      Strongly prefer in-person

Q19) How likely are you to continue using online resources or digital tools for skin condition management in the future?

·      Very likely

·      Somewhat likely

·      Neutral

·      Somewhat unlikely

·      Very unlikely

Q20) Do you think digital health tools and artificial intelligence will play a larger role in managing skin conditions in the future?

·      Yes

·      No

·      Unsure

Q21) What improvements would you like to see in online resources or digital tools for skin condition management? (optional question)

Long-answer text
